# Chest X-Ray Findings in COVID-19 Patients Presenting to Primary Care during the Peak of the First Wave of the Pandemic in Qatar: Their Association with Clinical and Laboratory Findings

**DOI:** 10.1155/2021/4496488

**Published:** 2021-10-27

**Authors:** Abdelwahed Abougazia, Ahmed Alnuaimi, Amal Mahran, Tamer Ali, Ahmed Khedr, Banan Qadourah, Ahmed Shareef, Soubhi Zitouni, Servet Kahveci, Barham Alqudah, Yasser Al Yassin, Mohamed Eldesoky, Ahmed Abdelmoneim, Reda Youssef

**Affiliations:** ^1^Radiology Department, Primary Health Care Corporation, Doha, Qatar; ^2^Pulmonology Department, Doha Clinic Hospital, Doha, Qatar; ^3^Clinical Imaging Department, Hamad Medical Corporation, Doha, Qatar

## Abstract

When managing coronavirus disease 2019 (COVID-19) patients, radiological imaging complements clinical evaluation and laboratory parameters. We aimed to assess the sensitivity of chest radiography findings in detecting COVID-19, describe those findings, and assess the association of positive chest radiography findings with clinical and laboratory findings. A multicentre, cross-sectional study was conducted involving all primary health care corporation-registered patients (2485 patients) enrolled over a 1-month period during the peak of the 2020 pandemic wave in Qatar. These patients had reverse transcription-polymerase chain reaction-confirmed COVID-19 and underwent chest radiography within 72 hours of the swab test. A positive result on reverse transcription-polymerase chain reaction was the gold standard for diagnosing COVID-19. The sensitivity of chest radiography was calculated. The airspace opacities were mostly distributed in the peripheral and lower lung zones, and most of the patients had bilateral involvement. Pleural effusion was detected in some cases. The risk of having positive chest X-ray findings increased with age, Southeast Asian nationality, fever, or a history of fever and diarrhoea. Patients with cardiac disease, obesity, hypertension, diabetes, and chronic kidney disease were at a higher risk of having positive chest X-ray findings. There was a statistically significant increase in the mean serum albumin, white blood cell count, neutrophil count, and serum C-reactive protein, hepatic enzymes, and total bilirubin with an increase in the radiographic severity score.

## 1. Introduction

At the end of 2019, the severe acute respiratory syndrome coronavirus-2 (SARS-CoV-2) spread from China to different parts of the world, and the ensuing pandemic was officially named coronavirus disease 19 (COVID-19). The disease can be asymptomatic or present with respiratory and/or systemic symptoms [1]. The gold standard diagnostic test for COVID-19 is the real-time reverse transcription-polymerase chain reaction- (RT-PCR-) based detection of the viral nucleic acids; however, RT-PCR has low sensitivity [2]. If RT-PCR is not available or if the results are negative in symptomaticCOVID-19 patients, chest imaging is considered a part of the screening procedure for suspected COVID-19 cases [3]. Radiological imaging complements clinical evaluation and laboratory parameters for managing COVID-19 patients [4]. Computed tomography (CT) is specific and more sensitive (95%) than chest X-ray (CXR) for diagnosing this disease, and CT was used in China during the peak of the first wave of the pandemic [5, 6]. The Fleischner Society stated that a CT scan is not suitable for screening or initial diagnosis of COVID-19 [7] because it requires time-consuming decontamination procedures to prevent the risk of cross-infection [8]. Therefore, CXR can be used instead of CT because of its extensive availability and easy and quick decontamination procedures. Few studies have compared the sensitivity of CXR and RT-PCR for detecting COVID-19 [9–14]; however, the association between CXR findings and clinical and laboratory findings has not been assessed adequately. The new standard operating procedures in the COVID-19 health centres of Qatar recommend a baseline CXR for all RT-PCR-positive COVID-19 patients. Studies have shown that the typical CXR findings of COVID-19 include bilateral peripheral and basal multifocal airspace opacities (ground-glass opacity (GGO) and consolidation) [9, 15]. Various patterns of CXR findings may be observed among the local COVID-19-positive patients. Recent studies on COVID-19 laboratory data showed an increase in serum levels of lactate dehydrogenase (LDH), C-reactive protein (CRP), and hepatic enzymes, with a marked decrease in lymphocytes. Studies have also shown that CRP and LDH have prognostic value, with a significant increase in patients with severe disease than in patients with mild disease [11, 16].

This study is aimed at assessing the sensitivity of CXR findings in detecting COVID-19, describing those findings, and assessing their association with laboratory and clinical findings. The study objectives were to (1) assess CXR sensitivity in COVID-19 patients in primary health care centres; (2) identify the major predictors for positive CXR (CXR+) findings and their association with sociodemographic characteristics, comorbidities, laboratory findings, and clinical findings; (3) describe CXR findings in COVID-19 patients presenting early to primary health care centres.

## 2. Materials and Methods

### 2.1. Methods

Qatar is a peninsular Arab country that operates a universal publicly funded health care system that is accessible to Qatari nationals and expatriates. The primary healthcare service in Qatar is delivered by the Primary Health Care Corporation (PHCC), which is the largest publicly funded primary care provider in the country. Currently, the PHCC operates 27 health centres. The current study is a multicentre cross-sectional descriptive study involving all PHCC registered patients with RT-PCR-confirmed COVID-19 diagnosis and a valid CXR performed within 72 h of the swab test. The study sample included all electronic health records that satisfied the abovementioned inclusion criteria obtained over a 1-month period during the peak of the 2020 pandemic wave in Qatar (from May 15, 2020, to June 14, 2020). A positive RT-PCR result was the gold standard for diagnosing COVID-19, irrespective of the symptoms. The variables that were requested from the data custodian of the PHCC were as follows: (1) the Visual Triage Checklist for COVID-19; (2) age, sex, and nationality of the patients to describe the demographics of the study population; (3) history of comorbidities for the following conditions: cardiac disease, hypertension, obesity (body mass index of ≥30 kg/m^2^), diabetes mellitus, liver disease, lung disease, oncologic history, and chronic kidney disease (CKD); (4) the available laboratory data that were completed within 72 h of the positive RT-PCR results—the laboratory data included albumin levels, white blood cell (WBC) count, lymphocyte count, CRP, creatine kinase levels, LDH, and liver enzymes; and (5) CXRs that were stored in the electronic health records system.

### 2.2. Image Acquisition and Analysis

CXRs were acquired as digital radiographs according to the local protocols. Each CXR was assessed by two radiologists with over 5 years of experience. In case of a disagreement, a final decision was made by a consultant radiologist with over 20 years of experience. CXRs were rated as positive or negative to assess the sensitivity of CXR compared to RT-PCR of nasopharyngeal and throat swabs—the gold standard. According to the Fleischner Society glossary, GGO, on CT images, appears as an area of increased attenuation in the lung with preserved bronchial and vascular markings; however, on chest radiographs, it is seen as a region of hazy lung radiopacity, in which the edges of the pulmonary vessels may be difficult to appreciate. Consolidation refers to a homogeneous opacification that obscures the airway walls and blood vessels. Reticulation refers to a collection of innumerable small opacities in a linear pattern [17].

In the case of positive CXR findings, the predominant pattern of CXR findings was documented as follows: the presence and distribution of airspace opacities (consolidation, GGOs, and reticular and nodular opacities). Regarding the distribution of lesions on CXRs, we considered halfway between the lateral edge of the lung and the hilum to divide lesions into peripheral predominant, perihilar predominant, or diffuse (neither peripheral nor perihilar). The laterality of the lesions is mentioned as right, left, or bilateral lungs. The zones involved are divided into upper, middle, or lower zone involvement. The upper zone extended from the apices of the lungs to the superior hilar markings, the middle zone extended from the superior hilar markings to the inferior hilar markings, and the lower zone extended from the inferior hilar markings to the costophrenic sulcus. Other features of CXR, such as pleural effusion and pneumothorax, were also assessed. The Radiographic Assessment of Lung Oedema score proposed by Warren et al. [18] was used to determine the severity score of each lung. The score is determined by the involvement of each lung in terms of consolidation or GGO, scored from 0 to 4 (score 0 = no involvement; 1 ≤ 25% involvement; 2 = 25–50% involvement; 3 ≥ 50–75% involvement; and 4 ≥ 75% involvement). The scores for each lung were summed to obtain the final COVID-19 radiographic severity score.

### 2.3. Statistical Analysis

The quantitative normally distributed variables are described as arithmetic means and measures of dispersion (standard deviation and standard error). The difference in the mean between more than two groups was assessed for statistical significance using analysis of variance. Categorical variables are presented as frequencies and percentages. The prevalence ratio (PR) was used to measure the strength of association between a dichotomous independent variable (a specific group compared with the reference group) and a dichotomous outcome variable (having a CXR+ finding). PR is the ratio between the prevalence of outcome (CXR+) among those with risk factors divided by the prevalence rate among those negative for the risk factor. The logarithm method was used to calculate the confidence intervals (CIs) for PR. For a PR with a 95% CI containing the null value of one, the association is not statistically significant at the 0.05 level of statistical significance. The multiple logistic regression model was used to predict the net (unconfounded) risk of having a positive outcome (CXR+ findings) for each explanatory variable included in the model.

## 3. Results and Discussion

### 3.1. Results

The baseline characteristics of the study population and the relative frequencies of the selected comorbid conditions are listed in Tables 1 and 2.

**Figure 1:**
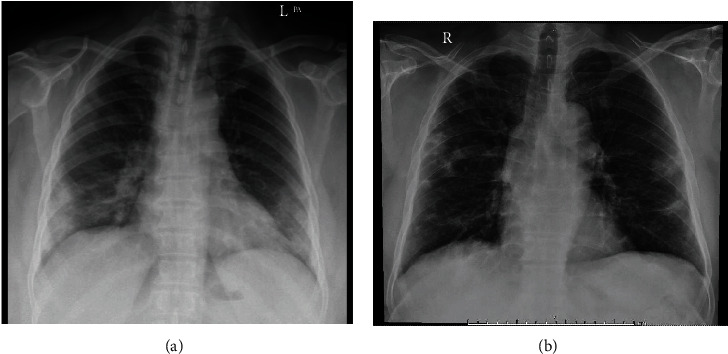
Chest x-rays of two patients (a and b). (a) Peripheral focal areas of ground glass opacities and consolidation at bilateral middle and lower lung zones. (b) Peripheral areas of ground glass opacities and consolidation at right upper, bilateral middle, and lower lung zones.

**Figure 2:**
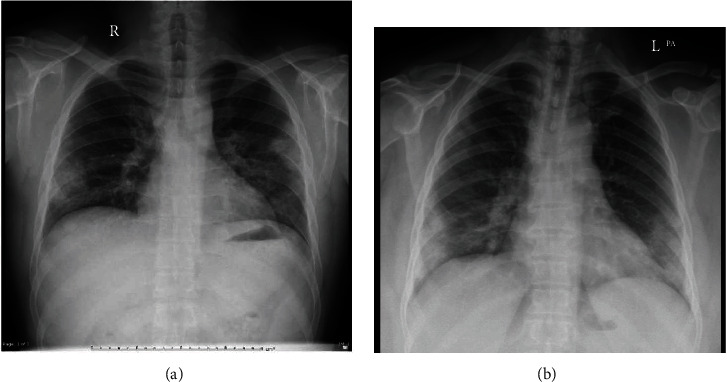
Chest x-rays of two patients (a and b). (a) Focal areas of ground glass opacities and consolidation at the peripheral areas of the middle and lower lung zones of both lungs with reticular interstitial thickening at the left middle and lower lung zones. (b) Peripheral focal areas of ground glass opacities and consolidation at bilateral middle and lower lung zones.

**Figure 3:**
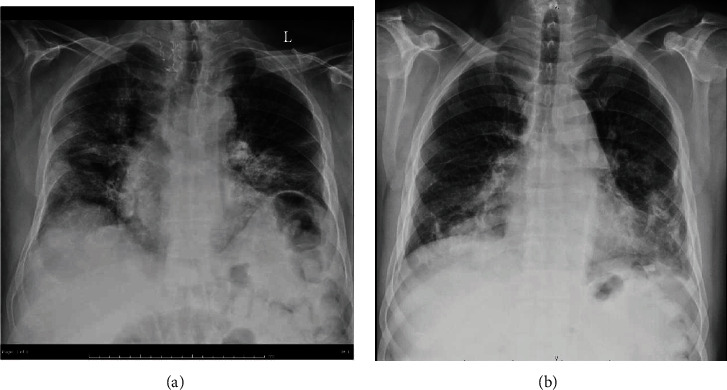
Chest x-rays of two patients (a and b). (a) Focal areas of ground glass opacities and consolidation with reticular interstitial thickening in peripheral and central regions of right upper, bilateral middle, and lower zones. (b) Focal areas of ground glass opacities and consolidation at left middle and bilateral lower lung zones with obliterated left lateral costophrenic recess suggesting pleural effusion.

Of the 2485 RT-PCR-confirmed COVID-19 patients, 417 were found to have a CXR+ finding suggestive of COVID-19. Considering RT-PCR as the gold standard for diagnosing COVID-19, the sensitivity of CXR was found to be 16.8% (95% CI: 15.3-18.3%) among the patients who presented to a primary health care setting. The airspace opacities found on CXRs were mostly distributed in the peripheral (69.5%) and lower (87.1%) lung zones. In addition, most patients had bilateral involvement (74%). Pleural effusion was detected in 10 patients (2.4%), with 2 patients having this sign as an isolated finding without an obvious parenchymal lung lesion on CXR. Pneumothorax was not detected in any of the patients. The CXR findings are summarized in Table 3 and Figures 1–3. The most common pattern of the parenchymal lung lesions was GGO (92.6%), followed by consolidation (22.8%), reticular interstitial thickening (21.8%), and nodular pattern (2.4%).

As shown in Table 4, compared with young adults, the PR (risk of having CXR+) significantly increased in the older adult group (40-64 years) by 2.52 times, and in the middle age/elderly group (>65 years) by 4.59 times. In contrast, the younger age groups had a lower risk of COVID-19; however, their risk estimates were not statistically significant. Among the nationality categories, the Southeast Asian nationality groups were associated with a significant increase in the risk of having CXR+ (2.55 times) compared with the other groups (miscellaneous nationality).

The clinical features were assessed only for a subset of 625 cases (Supplementary file, Table 1). The most frequently reported symptoms were fever or history of fever (57.6%), followed by cough (42.4%), sore throat (34.2%), headache (14.1%), runny nose (9.6%), shortness of breath (5.8%), arthralgia (1.9%), diarrhoea (1.8%), and neck pain (0.2%). Only fever and diarrhoea were associated with a statistically significant increase in the risk of having CXR+ findings (Supplementary file, Table 2. Fever or history of fever increased this risk by 1.66, while diarrhoea increased the risk of the outcome by 2.29 times.

Several comorbidities were tested for their ability to predict CXR+ findings (Supplementary file, Table 3). Cardiac disease, obesity, hypertension, diabetes, and CKD were associated with a significantly increased risk of having a CXR+ (1.96 times, 2.44 times, 1.37 times, 2.54 times, and 2.36 times, respectively).

The frequency distribution of the study sample by radiographic scoring of the parenchymal lung lesions of the 415 cases with a CXR+ finding was determined (Supplementary file, Table 4). The most common scores were mild, including types 1 (45.1%) and 2 (40.5%). Moderate severity scores ranging between 3 and 5 were found in 13.5% of cases, while severe cases with a severity score of 6 were found in 0.9% of cases.

The difference in the mean of the selected laboratory test values and the total severity score (TSS) of CXR (classified into three categories) is shown in the Supplementary file (Table 5). There was an obvious and statistically significant increase in the mean serum albumin level, blood WBC count, neutrophil count, serum CRP, serum alanine aminotransferase (ALT), serum aspartate aminotransferase (AST), and serum total bilirubin with an increase in the radiographic severity score (a change in total pulmonary radiographic scoring categories from no pulmonary involvement to low score and high score categories).

The multiple logistic regression model was used to predict the net (unconfounded) risk of having a CXR+ COVID-19 finding by selected comorbidities after adjusting for each comorbidity in addition to age, sex, and nationality. Using the backward selection method, only hypertension and diabetes significantly increased the risk of a positive outcome by 28% and 72%, respectively (Supplementary file Table 6).

The multiple logistic regression model was used to predict the net (unconfounded) risk of having a positive COVID-19 CXR finding based on selected clinical features after adjusting for each comorbidity in addition to age, sex, and nationality. Using the backward selection method, only “fever or history of fever” and “shortness of breath” significantly increased the risk of a positive outcome by 2 and 2.3 times, respectively (Supplementary file, Table 7).

## 4. Discussion

Only few papers in the literature have assessed CXR sensitivity in COVID-19 patients who presented early to a primary health care centre. Our study showed a CXR sensitivity of 16.8% (95% CI: 15.3-18.3%) in identifying abnormalities associated with a positive COVID-19 diagnosis.

We compared our results with those reported by other authors [9–14, 19, 20]. The previously reported sensitivities ranged from 11.4% (Rousan et al.) to 89% (Schiaffano et al.), while the other reported values were 41.7% (Weinstock et al.), 57.0% (Ippolito et al.), 59.1% (Guan et al.), 61.2% (Gatti et al.), 68.1% (Cozzi et al.), and 68.8% (Wong et al.). Rousan et al. [20] and Weinstock et al. [10] reported a relatively low sensitivity due to the early admission of patients with low-grade pathology.

Schiaffano et al. [14] reported a relatively high sensitivity as their study included hospitalized patients in endemic areas with high-grade pathology. Ng et al. [21] reported that CXR was not sensitive in the early stages of lung disease. The lower value reported in our study is because our patients (presenting to a primary health care setting) were suspected to have COVID-19 and were screened initially with an RT-PCR throat swab at the early stages of the disease (within 3 days after a positive swab result) with low-grade pathology.

Our study showed that GGO was the most common CXR finding in patients with peripheral lower zone and bilateral lung involvement. Our findings are consistent with those reported in previous CXR and CT studies [19, 21–25]. Pleural effusion was not common and was reported in only 10 (2.4%) of our patients [21, 26].

Regarding the clinical data, the most frequently reported symptoms were fever or history of fever (57.6%), followed by cough (42.4%), sore throat (34.2%), headache (14.1%), runny nose (9.6%), shortness of breath (5.8%), arthralgia (1.9%), diarrhoea (1.8%), and neck pain (0.2%). A statistically significant increase in the risk of CXR+ findings was noted in patients with fever or diarrhoea. Fever or history of fever increased the risk by 66%, while diarrhoea increased the risk of the outcome by 2.29 times. Diarrhoea was an uncommon symptom in previous studies [1, 11, 24]. Rousan et al. [20] reported a significant association between CXR findings and symptoms (*P* = 0.005), and most (92.3%) patients with CXR+ findings were symptomatic.

The CXR scoring system provides a semiquantitative tool to assess lung abnormalities and help clinicians stratify the disease risk [9]. A score, ranging from 0 to 4, was given to each lung in our study according to the extent of lung involvement (score 0 = no involvement; 1 ≤ 25% involvement; 2 = 25 − 50% involvement; 3 = 50 − 75% involvement; 4 ≥ 75% lung involvement). By summing the scores of both the lungs, the TSS, ranging from 0 to 8, was calculated.

The frequency distribution of the study sample by radiographic scoring of the parenchymal lung lesions of the 415 cases with CXR+ findings was determined.

The most common scores were mild, including types 1 (45.1%) and 2 (40.5%). A moderate severity score was observed in 13.5% of cases (ranging from 3 to 5), while 0.9% of cases had a severe severity score (6). Wong et al. [9] reported that 41% of cases had mild TSS (1-2), while 20% and 8% had moderate (TSS = 3 − 4) and severe (TSS = 5 − 6) scores, respectively. In agreement with Wong et al. [9], no patient had a TSS > 6 in our study. In a previous study, the CXR severity score helped clinicians stratify the disease risk and identify patients who needed hospital admission and intubation [27].

The difference in the mean of the selected laboratory test values and the TSS of CXR (classified into three categories) were assessed. There was an obvious and statistically significant increase in the mean serum albumin, WBC count, neutrophil count, serum CRP, serum ALT, serum AST, and serum total bilirubin with an increase in radiographic TSS (a change in the total pulmonary radiographic scoring categories from no pulmonary involvement to low score and high score categories).

Recent studies have shown that a few laboratory features are associated with severe degrees of COVID-19 [1, 11, 16]. In a study of 1099 patients, Guan et al. [11] detected a significant increase in LDH and CRP in patients with severe disease than in patients with mild disease. High CRP levels in COVID-19 patients (due to inflammatory reaction and tissue destruction) indicate a more severe illness and worse prognosis with lung damage [28]. According to Gatti et al. [19], CRP is a major predictor of CXR+.

Our study (in agreement with the study by Gatti et al.) suggests that baseline CXR and laboratory evaluations can identify severe COVID-19 cases when RT-PCR tests are limited. The results of our study (in agreement with the results of the study by Gatti et al.) showed that most of the early screened COVID-19 patients without a severe increase in CRP had a negative CXR.

The large sample size (2485 COVID-19 patients) is a major strength of our study. The limitations of our study included the assessment of CXR sensitivity without comparison of the specificity and predictive values with a control group. Second, there was no correlation between the CXR outcome and the clinical outcome, which should be the goal of further prospective studies.

## 5. Conclusions

Our study described the baseline CXR findings of 2485 positive COVID-19 patients. The CXR sensitivity was 16.8%, and GGO was the most common CXR finding with peripheral lower zone and bilateral lung involvement. The risk of having CXR features of pulmonary involvement increased with age, Southeast Asian nationality, and fever or a history of fever and diarrhoea as clinical features. In addition, having selected comorbidities, namely, cardiac disease, obesity, hypertension, diabetes, and CKD, predicts a higher risk of CXR findings. There was a statistically significant increase in the mean serum albumin level, WBC count, neutrophil count, serum CRP, serum ALT, serum AST, and serum total bilirubin with an increase in the radiographic severity score.

## Figures and Tables

**Table 1:** Description of the study sample.

	*N*	%
Gender		
Female	816	32.8
Male	1669	67.2
Total	2485	100.0
Age group (years)		
Preschool children (<5)	37	1.5
School age children (5-9)	63	2.5
Teenagers (10-17)	108	4.3
Young adults (18-39)	1267	51.0
Older adults (40-64)	933	37.5
Middle age/elderly (65+)	77	3.1
Total	2485	100.0
Nationality categories		
Northern Africa	489	19.7
South-Eastern Asia	173	7.0
Southern Asia	1127	45.4
Western Asia	621	25.0
Other (miscellaneous) nationality	75	3.0
Total	2485	100.0

**Table 2:** The relative frequency of selected comorbid conditions.

Comorbid conditions (*N* = 2485)	*N*	%
Obesity (BMI ≥ 30 kg/m^2^)	518	20.8
Diabetes	481	19.4
Hypertension	434	17.5
Asthma	200	8.0
Cardiac disease	75	3.0
Chronic kidney disease	36	1.4
Cancer	24	1.0
Chronic lung disease	4	0.2

**Table 3:** The relative frequency of selected radiographic findings.

Positive COVID-19 CXR findings (*N* of total with positive COVID-19 CXR finding = 417)	*N*	%
Ground glass opacity	386	92.6
Consolidation	95	22.8
Reticular interstitial thickening	91	21.8
Nodular	10	2.4
Pleural effusion	10	2.4
Upper lung zone	44	10.6
Middle lung zone	200	48.0
Lower lung zone	363	87.1
Peripheral pulmonary lesions	290	69.5
Central (perihilar) pulmonary lesions	16	3.8
Diffuse (neither peripheral nor central) pulmonary lesions	107	25.7

Note: none had pneumothorax.

**Table 4:** The relative frequency for showing any positive COVID-19 CXR finding by sociodemographic variables.

	Any positive CXR finding						PR	95% confidence interval PR
	Negative		Positive		Total			
	*N*	%	*N*	%	*N*	%		
Gender								
Female	689	84.4	127	15.6	816	100	Ref	
Male	1379	82.6	290	17.4	1669	100	1.12	(0.93-1.36)
Age group (years)								
Preschool children (<5)	34	91.9	3	8.1	37	100	0.79	(0.26-2.37)
School age children (5-9)	61	96.8	2	3.2	63	100	0.31	(0.08-1.22)
Teenagers (10-17)	101	93.5	7	6.5	108	100	0.64	(0.31-1.33)
Young adults (18-39)	1138	89.8	129	10.2	1267	100	Ref	
Older adults (40-64)	693	74.3	240	25.7	933	100	2.52	(2.07-3.07)
Middle age/elderly (65+)	41	53.2	36	46.8	77	100	4.59	(3.44-6.13)
Nationality categories								
Other (miscellaneous) nationality	68	90.7	7	9.3	75	100	Ref	
Northern Africa	401	82	88	18	489	100	1.94	(0.93-4.03)
South-Eastern Asia	132	76.3	41	23.7	173	100	2.55	(1.2-5.42)
Southern Asia	929	82.4	198	17.6	1127	100	1.89	(0.92-3.87)
Western Asia	538	86.6	83	13.4	621	100	1.44	(0.69-3)

## Data Availability

All data generated or analyzed during this study are included in this published article and supplementary file.

## Abbreviations

ALT: Alanine aminotransferase
AST: Aspartate aminotransferase
BMI: Body mass index
CI: Confidence interval
CK: Creatine kinase
CKD: Chronic kidney disease
COVID-19: Coronavirus disease 2019
CRP: C-reactive protein
CT: Computed tomography
CXR: Chest X-ray
CXR+: Positive CXR
CXR-: Negative CXR
EHR: Electronic health records
GGO: Ground-glass opacification/opacity
HIM: Health information management
LDH: Lactate dehydrogenase
*N*: Number
NS: Nonsignificant
*P*: *P* value
PHCC: Primary Health Care Corporation
PR: Prevalence ratio
Ref: Reference
RIS PACS: The radiology information system and picture archiving and communications system
RT-PCR: Real-time reverse transcription-polymerase chain reaction
SD: Standard deviation
SE: Standard error
TSS: Total severity score
WBC: White blood cells.

## Supplementary Materials

Supplementary Materials Table 1: frequency distribution of the study sample by clinical features. Table 2: the relative frequency for showing any positive COVID-19 CXR finding by clinical features. Table 3: the relative frequency for showing any positive COVID-19 CXR finding by comorbid conditions. Table 4: frequency distribution of the study sample by radiographic scoring. Table 5: the mean of selected laboratory test values by severity of COVID19 CXR findings. Table 6: multiple logistic regression model for predicting the risk of having a positive CXR finding by comorbidities and sociodemographic variables (age, gender, and nationality). Table 7: multiple logistic regression model for predicting the risk of having a positive CXR finding by clinical features and sociodemographic variables (age, gender, and nationality).
