# Supplementary material for: Chest X-Ray Findings in COVID-19 Patients Presenting to Primary Care during the Peak of the First Wave of the Pandemic in Qatar: Their Association with Clinical and Laboratory Findings
Source: Pulm Med. 2021 Oct 27;2021:4496488. doi: 10.1155/2021/4496488 (PMC8553512; doi:10.1155/2021/4496488)
Supplement: Supplementary Materials — Table 1: frequency distribution of the study sample by clinical features. Table 2: the relative frequency for showing any positive COVID-19 CXR finding by clinical features. Table 3: the relative frequency for showing any positive COVID-19 CXR finding by comorbid conditions. Table 4: frequency distribution of the study sample by radiographic scoring. Table 5: the mean of selected laboratory test values by severity of COVID19 CXR findings. Table 6: multiple logistic regression model for predicting the risk of having a positive CXR finding by comorbidities and sociodemographic variables (age, gender, and nationality). Table 7: multiple logistic regression model for predicting the risk of having a positive CXR finding by clinical features and sociodemographic variables (age, gender, and nationality). [file 4496488.f1.docx]

Table 1: Frequency distribution of the study sample by clinical features.

| **Clinical features (N=625)** | **N** | **%** | **95% confidence interval** |
| --- | --- | --- | --- |
| Fever or History of Fever | 360 | 57.6 | (53.7 to 61.5) |
| Cough | 265 | 42.4 | (38.5 to 46.3) |
| Sore Throat | 214 | 34.2 | (30.5 to 37.9) |
| Headache | 88 | 14.1 | (11.4 to 16.8) |
| Runny Nose | 60 | 9.6 | (7.3 to 11.9) |
| Shortness of Breath | 36 | 5.8 | (4 to 7.6) |
| Arthralgia | 12 | 1.9 | (0.8 to 3) |
| Diarrhea | 11 | 1.8 | (0.8 to 2.8) |
| Neck Pain | 1 | 0.2 | (-0.2 to 0.6) |

Table 2: The relative frequency for showing any positive COVID-19 CXR finding by clinical features.

|  | **Any positive CXR finding** | | | | | |  | **95% Confidence Interval PR** |
| --- | --- | --- | --- | --- | --- | --- | --- | --- |
|  | **Negative** | | **Positive** | | **Total** | |  |  |
|  | **N** | **%** | **N** | **%** | **N** | **%** | **PR** |  |
| Fever or History of Fever |  |  |  |  |  |  |  |  |
| Negative | 226 | 85.3 | 39 | 14.7 | 265 | 100 | Ref |  |
| Positive | 272 | 75.6 | 88 | 24.4 | 360 | 100 | 1.66 | (1.18 - 2.34) |
|  |  |  |  |  |  |  |  |  |
| Sore Throat |  |  |  |  |  |  |  |  |
| Negative | 322 | 78.3 | 89 | 21.7 | 411 | 100 | Ref |  |
| Positive | 176 | 82.2 | 38 | 17.8 | 214 | 100 | 0.82 | (0.58 - 1.15) |
|  |  |  |  |  |  |  |  |  |
| Cough |  |  |  |  |  |  |  |  |
| Negative | 296 | 82.2 | 64 | 17.8 | 360 | 100 | Ref |  |
| Positive | 202 | 76.2 | 63 | 23.8 | 265 | 100 | 1.34 | (0.98 - 1.83) |
|  |  |  |  |  |  |  |  |  |
| Shortness of Breath |  |  |  |  |  |  |  |  |
| Negative | 473 | 80.3 | 116 | 19.7 | 589 | 100 | Ref |  |
| Positive | 25 | 69.4 | 11 | 30.6 | 36 | 100 | 1.55 | (0.92 - 2.6) |
|  |  |  |  |  |  |  |  |  |
| Headache |  |  |  |  |  |  |  |  |
| Negative | 427 | 79.5 | 110 | 20.5 | 537 | 100 | Ref |  |
| Positive | 71 | 80.7 | 17 | 19.3 | 88 | 100 | 0.94 | (0.59 - 1.49) |
|  |  |  |  |  |  |  |  |  |
| Runny Nose |  |  |  |  |  |  |  |  |
| Negative | 447 | 79.1 | 118 | 20.9 | 565 | 100 | Ref |  |
| Positive | 51 | 85 | 9 | 15 | 60 | 100 | 0.72 | (0.39 - 1.34) |
|  |  |  |  |  |  |  |  |  |
| Neck Pain |  |  |  |  |  |  |  |  |
| Negative | 497 | 79.6 | 127 | 20.4 | 624 | 100 | Ref |  |
| Positive | 1 | 100 | 0 | 0 | 1 | 100 | ** | ** |
|  |  |  |  |  |  |  |  |  |
| Arthralgia |  |  |  |  |  |  |  |  |
| Negative | 488 | 79.6 | 125 | 20.4 | 613 | 100 | Ref |  |
| Positive | 10 | 83.3 | 2 | 16.7 | 12 | 100 | 0.82 | (0.23 - 2.93) |
|  |  |  |  |  |  |  |  |  |
| Diarrhea |  |  |  |  |  |  |  |  |
| Negative | 492 | 80.1 | 122 | 19.9 | 614 | 100 | Ref |  |
| Positive | 6 | 54.5 | 5 | 45.5 | 11 | 100 | 2.29 | (1.18 - 4.46) |

Table 3: The relative frequency for showing any positive COVID-19 CXR finding by co-morbid conditions.

|  | **Any positive CXR finding** | | | | | |  | **95% Confidence Interval PR** |
| --- | --- | --- | --- | --- | --- | --- | --- | --- |
|  | **Negative** | | **Positive** | | **Total** | |  |  |
|  | **N** | **%** | **N** | **%** | **N** | **%** | **PR** |  |
| Cardiac disease |  |  |  |  |  |  |  |  |
| Negative | 2017 | 83.7 | 393 | 16.3 | 2410 | 100 | Ref |  |
| Positive | 51 | 68 | 24 | 32 | 75 | 100 | 1.96 | (1.39 - 2.76) |
| Hypertension |  |  |  |  |  |  |  |  |
| Negative | 1776 | 86.6 | 275 | 13.4 | 2051 | 100 | Ref |  |
| Positive | 292 | 67.3 | 142 | 32.7 | 434 | 100 | 2.44 | (2.05 - 2.9) |
| Obesity (BMI ≥ 30 Kg/m2) |  |  |  |  |  |  |  |  |
| Negative | 1661 | 84.4 | 306 | 15.6 | 1967 | 100 | Ref |  |
| Positive | 407 | 78.6 | 111 | 21.4 | 518 | 100 | 1.37 | (1.13 - 1.66) |
| Diabetes |  |  |  |  |  |  |  |  |
| Negative | 1745 | 87.1 | 259 | 12.9 | 2004 | 100 | Ref |  |
| Positive | 323 | 67.2 | 158 | 32.8 | 481 | 100 | 2.54 | (2.14 - 3.01) |
| Asthma |  |  |  |  |  |  |  |  |
| Negative | 1909 | 83.5 | 376 | 16.5 | 2285 | 100 | Ref |  |
| Positive | 159 | 79.5 | 41 | 20.5 | 200 | 100 | 1.24 | (0.93 - 1.65) |
| Chronic Lung Disease |  |  |  |  |  |  |  |  |
| Negative | 2065 | 83.2 | 416 | 16.8 | 2481 | 100 | Ref |  |
| Positive | 3 | 75 | 1 | 25 | 4 | 100 | 1.49 | (0.27 - 8.15) |
| Cancer |  |  |  |  |  |  |  |  |
| Negative | 2047 | 83.2 | 414 | 16.8 | 2461 | 100 | Ref |  |
| Positive | 21 | 87.5 | 3 | 12.5 | 24 | 100 | 0.74 | (0.26 - 2.14) |
| Chronic Kidney Disease |  |  |  |  |  |  |  |  |
| Negative | 2046 | 83.5 | 403 | 16.5 | 2449 | 100 | Ref |  |
| Positive | 22 | 61.1 | 14 | 38.9 | 36 | 100 | 2.36 | (1.55 - 3.59) |

Table 4: Frequency distribution of the study sample by radiographic scoring.

|  | **N** | **%** |
| --- | --- | --- |
| Right lung Radiographic scoring |  |  |
| No involvement | 2163 | 87.0 |
| <25% of lung | 269 | 10.8 |
| 25-49% | 45 | 1.8 |
| 50-74% | 8 | 0.3 |
| Total | 2485 | 100.0 |
| Left lung Radiographic scoring |  |  |
| No involvement | 2176 | 87.6 |
| <25% of lung | 258 | 10.4 |
| 25-49% | 44 | 1.8 |
| 50-74% | 7 | 0.3 |
| Total | 2485 | 100.0 |
| Total pulmonary radiographic scoring* |  |  |
| No involvement | 2070 | 83.3 |
| 1 | 187 | 7.5 |
| 2 | 168 | 6.8 |
| 3 | 28 | 1.1 |
| 4 | 21 | 0.8 |
| 5 | 7 | 0.3 |
| 6 | 4 | 0.2 |
| Total | 2485 | 100.0 |

**Note: Two isolated Pleural effusion cases received no scoring.**

Table 5: The mean of selected laboratory test values by severity of COVID19 CXR findings.

|  |  | **Total pulmonary radiographic scoring-categories** | | |  |
| --- | --- | --- | --- | --- | --- |
|  |  | **No pulmonary involvement** | **Low score (1-2)** | **High score (3-6)** | **P** |
| Serum Albumin (gm/L) | Mean ± SD | 45.3 ± 3.38 | 43.2 ± 3.53 | 40.9 ± 3.43 | <0.001 |
| Blood WBC count (x10^3/Ul) | Mean ± SD | 5.6 ± 1.92 | 5.6 ± 1.84 | 6.6 ± 2.57 | <0.001 |
| Blood Hgb (gm/dL) | Mean ± SD | 14.1 ± 1.76 | 13.7 ± 1.65 | 13.9 ± 1.45 | 0.63[NS] |
| Lymphocyte’s count (x10^3/uL) | Mean ± SD | 2 ± 0.9 | 1.8 ± 0.68 | 1.7 ± 0.74 | 0.002 |
| Neutrophil count (x10^3/uL) | Mean ± SD | 3 ± 1.46 | 3.3 ± 1.55 | 4.4 ± 2.45 | <0.001 |
| Platelet’s count (x10^3/uL) | Mean ± SD | 244 ± 72.17 | 236.1 ± 80.41 | 245.4 ± 93.53 | 0.89[NS] |
| Serum CRP (mg/L) | Mean ± SD | 8 ± 14.98 | 29.6 ± 39.17 | 77.8 ± 66.67 | <0.001 |
| Serum ALT (U/L) | Mean ± SD | 32.9 ± 26.93 | 36.1 ± 26.43 | 41.2 ± 23.13 | 0.021 |
| Serum AST (U/L) | Mean ± SD | 30.5 ± 17.13 | 34.8 ± 23.5 | 44.4 ± 25.2 | <0.001 |
| Serum Total Bilirubin (umol/L) | Mean ± SD | 9.1 ± 5 | 9 ± 5.13 | 11.2 ± 6.73 | 0.003 |
| Serum Total Protein(gm/L) | Mean ± SD | 77.1 ± 5.06 | 75.6 ± 5.45 | 75 ± 4.5 | 0.002 |
| Serum Sodium (mmol/L) | Mean ± SD | 139.5 ± 2.69 | 138.7 ± 3.22 | 136.9 ± 5.13 | <0.001 |
| Serum Potassium (mmol/L) | Mean ± SD | 4.3 ± 0.44 | 4.3 ± 0.51 | 4.3 ± 0.48 | 0.66[NS] |
| Blood Urea (mmol/L) | Mean ± SD | 4.2 ± 1.36 | 4.4 ± 1.84 | 4.5 ± 1.69 | 0.06[NS] |
| Serum Creatinine (umol/L) | Mean ± SD | 71.9 ± 22.94 | 75.4 ± 27.72 | 76.6 ± 17.19 | 0.14[NS] |

Table 6: Multiple logistic regression model for predicting the risk of having a positive CXR finding by comorbidities and sociodemographic variables (age, gender and nationality).

| **A) Method=Enter** | **Adjusted OR** | **95% confidence interval OR** | **P** |
| --- | --- | --- | --- |
| Age group (years) compared to young adults (18-39) |  |  | <0.001 |
| Preschool children (<5) | 0.81 | (0.24 to 2.69) | 0.73[NS] |
| School age children (5-9) | 0.3 | (0.07 to 1.25) | 0.1[NS] |
| Teenagers (10-17) | 0.71 | (0.32 to 1.57) | 0.4[NS] |
| Older adults (40-64) | 2.32 | (1.79 to 3.01) | <0.001 |
| Middle age/elderly (65+) | 5.01 | (2.89 to 8.67) | <0.001 |
|  |  |  |  |
| Male gender compared to female | 1.15 | (0.89 to 1.48) | 0.3[NS] |
|  |  |  |  |
| Nationality categories compared other (miscellaneous) category |  |  | 0.001 |
| Northern Africa | 1.86 | (0.8 to 4.3) | 0.15[NS] |
| South-eastern Asia | 3.09 | (1.27 to 7.5) | 0.013 |
| Southern Asia | 1.77 | (0.78 to 4.02) | 0.17[NS] |
| Western Asia | 1.24 | (0.53 to 2.86) | 0.62[NS] |
|  |  |  |  |
| Co-morbidities |  |  |  |
| Cardiac disease | 0.94 | (0.55 to 1.63) | 0.84[NS] |
| Hypertension | 1.33 | (0.98 to 1.82) | 0.07[NS] |
| Obesity (BMI ≥ 30 Kg/m2) | 1.09 | (0.81 to 1.45) | 0.57[NS] |
| Diabetes | 1.69 | (1.25 to 2.27) | <0.001 |
| Asthma | 1.23 | (0.83 to 1.83) | 0.3[NS] |
| Chronic Lung Disease | 0.51 | (0.05 to 5.32) | 0.57[NS] |
| Cancer | 0.35 | (0.1 to 1.26) | 0.11[NS] |
| Chronic Kidney Disease | 1.25 | (0.6 to 2.63) | 0.55[NS] |
| Constant | 0.06 |  | <0.001 |

**P (Model) < 0.001**

**Overall predictive accuracy = 83.2%**

| **B) Method=Backward step** | **Adjusted OR** | **95% confidence interval OR** | **P** |
| --- | --- | --- | --- |
| Hypertension | 1.38 | (1.02 to 1.86) | 0.037 |
| Diabetes | 1.72 | (1.28 to 2.3) | <0.001 |

**P (Model) < 0.001**

**Overall predictive accuracy = 83.1%**

Table 7: Multiple logistic regression model for predicting the risk of having a positive CXR finding by clinical features and sociodemographic variables (age, gender and nationality).

| **A) Method=Enter** | **Adjusted OR** | **95% confidence interval OR** | **P** |
| --- | --- | --- | --- |
| Age group (years) compared to young adults (18-39) |  |  | <0.001 |
| Preschool children (<5) | <0.001 | (0 to 0) | 1[NS] |
| School age children (5-9) | 0.87 | (0.1 to 7.64) | 0.9[NS] |
| Teenagers (10-17) | <0.001 | (0 to 0) | 1[NS] |
| Older adults (40-64) | 2.5 | (1.62 to 3.87) | <0.001 |
| Middle age/elderly (65+) | 6.69 | (2.2 to 20.33) | <0.001 |
|  |  |  |  |
| Male gender compared to female | 0.99 | (0.59 to 1.66) | 0.97[NS] |
|  |  |  |  |
| Nationality categories compared other (miscellaneous) category |  |  | 0.07[NS] |
| Northern Africa | 3.91 | (0.47 to 32.67) | 0.21[NS] |
| South-eastern Asia | 7.66 | (0.83 to 70.87) | 0.07[NS] |
| Southern Asia | 3.1 | (0.38 to 25.04) | 0.29[NS] |
| Western Asia | 2.24 | (0.27 to 18.7) | 0.46[NS] |
|  |  |  |  |
| Clinical features |  |  |  |
| Fever or History of Fever | 1.97 | (1.24 to 3.13) | 0.004 |
| Sore Throat | 0.76 | (0.48 to 1.2) | 0.24[NS] |
| Cough | 1.09 | (0.71 to 1.68) | 0.69[NS] |
| Shortness of Breath | 2.29 | (1.01 to 5.21) | 0.047 |
| Headache | 0.98 | (0.53 to 1.79) | 0.95[NS] |
| Runny Nose | 0.8 | (0.36 to 1.77) | 0.58[NS] |
| Neck Pain | 0 | (0 to 0) | 1[NS] |
| Arthralgia | 0.66 | (0.14 to 3.25) | 0.61[NS] |
| Diarrhea | 2.48 | (0.69 to 8.91) | 0.16[NS] |
| Constant | 0.03 |  | 0.002 |

**P (Model) < 0.001**

**Overall predictive accuracy = 80.6%**

| **B) Method=Backward step** | **Adjusted OR** | **95% confidence interval OR** | **P** |
| --- | --- | --- | --- |
| Fever or History of Fever | 2 | (1.28 to 3.12) | 0.002 |
| Shortness of Breath | 2.3 | (1.05 to 5.1) | 0.037 |

**P (Model) < 0.001**

**Overall predictive accuracy = 80%**
